# Cetuximab response in CRC patient-derived xenografts seems predicted by an expression based RAS pathway signature

**DOI:** 10.18632/oncotarget.10499

**Published:** 2016-07-08

**Authors:** Sheng Guo, Dawei Chen, Xuesong Huang, Jie Cai, Jean-Pierre Wery, Qi-Xiang Li

**Affiliations:** ^1^ Division of Translational Oncology, Crown Bioscience, Inc., Santa Clara, CA 95054, USA; ^2^ State Key Laboratory of Natural and Biomimetic Drugs, Peking University, Beijing, 100191, China

**Keywords:** PDX, Erbitux, expression signature, patient stratification, biomarker

## Abstract

Cetuximab is an approved treatment for metastatic colorectal carcinoma (mCRC) with codon 12/13-KRAS mutations, recently questioned for its validity, and alternative mutation-based biomarkers were proposed. We set out to investigate whether an expression signature can also predict response by utilizing a cetuximab mouse clinical trial (MCT) dataset on a cohort of 25 randomly selected EGFR^+^ CRC patient-derived xenografts (PDXs). While we found that the expression of EGFR and its ligands are not predictive of the cetuximab response, we tested a published RAS pathway signature, a 147-gene expression signature proposed to describe RAS pathway activity, against this MCT dataset. Interestingly, our study showed that the observed cetuximab activity has a strong correlation with the RAS pathway signature score, which was also demonstrated to have a certain degree of correlation with a historic clinical dataset. Altogether, the independent validations in unrelated datasets from independent cohort of CRCs strongly suggest that RAS pathway signature may be a relevant expression signature predictive of CRC response to cetuximab. Our data seem to suggest that an mRNA expressing signature may also be developed as a predictive biomarker for drug response, similarly to genetic mutations.

## INTRODUCTION

EGFR-targeting monoclonal antibodies, such as cetuximab (Erbitux^®^) [1, 2], are important standard of care (SOC) targeted therapies for treating metastatic colorectal carcinoma (mCRC), excluding those with KRAS mutations at codons 12 and 13, and offer clinical benefit for a subset of mCRC patients [3]. However, only 35~50% of wild-type KRAS CRC patients responded to cetuximab [2, 4] and only ~10% of mCRC patients respond to cetuximab monotherapy as a second line treatment [5]. Importantly, several reports have suggested that the “KRAS 12/13 rule” is not an optimal criterion for the clinical use of cetuximab for treating CRC [2], but an alternative gene mutation signature might be more predictive. First, a recent retrospective analysis of multiple phase-III trials unexpectedly concluded that patients with KRAS codon 13 mutation (G13D) seem to benefit from the treatment [6], and our recent observation on a mouse clinical trial (MCT) using a cohort of randomly selected CRC patient-derived xenografts (PDXs) also clearly supported the same conclusion [7, 8]. Second, a number of new alternative mutation based signatures have been proposed to be better predictive of response to cetuximab [7, 8], including the activating mutations [2] of EGFR and BRAF (*e.g.* V600E) [2, 9], activation of ERBB2 signaling [10], KRAS mutations [2, 4, 11], PDGFRA and MAP2K1 [8], and composite mutation signatures of specific sets of oncogene mutation alleles. In practice, caution has been advised on the use of cetuximab in some KRAS-12/13 wild type patients.

There are many genetic and epigenetic properties of cancers that can be potentially monitored for predicting drug responses. However, it seems that only genetic alterations in DNA have some success in general. For example, EGFR activating mutation for EGFR-TKIs in lung cancer [12–14], c-met amplification in lung cancer for MET-TKI [13], ALK fusion for ALK-TKI in lung cancer [15], HER2 amplification in gastric and breast cancers for Herceptin^®^, *etc*. In contrast, there are few epigenetic predictive biomarkers, *e.g*. gene expression in mRNA, that were validated for reasons to be elucidated. Several reports have suggested some epigenetic alterations predictive of cetuximab response, either positively or negatively, including gene amplification/over-expression of EGFR or its ligands, epiregulin (EREG) and amphiregulin (AREG) [3, 16]. With conflicting and inconclusive observations so far, it remains a challenge to predict the responders using expression based criteria.

We have explored predictive biomarkers for CRC-cetuximab response using MCT datasets, not only those based on genetic alterations [7], but also those based on gene expression. The present study tested the Loboda RAS pathway signature for its correlation with response in our MCT of a cohort of 25 EGFR-expressing CRC PDXs [7], and revealed that the RAS pathway signature [17] seems to predict CRC response to cetuximab, consistent with recent clinical data [17].

## RESULTS

### Expression of EGFR and its ligands seems to have little influence on cetuximab response in CRC-PDX

**Figure 1:**
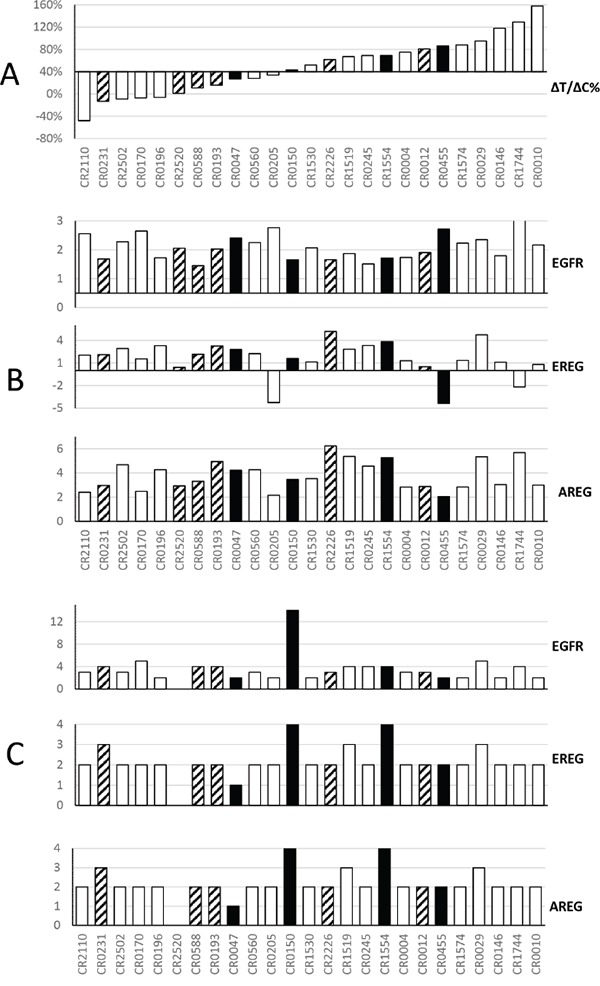
Summary of cetuximab activity and corresponding genomic profiles of EGFR, Epiregulin and Amphiregulin in a cohort of CRC-PDXs **Panel A.** Waterfall plot of the ΔT/ΔC% of PDX: cetuximab vs. control, ranked from responders on the left to non-responders on the right (Y-axis: ΔT/ΔC). The open bars are wild type KRAS, the solid bar are KRAS G12D/V/C mutants, and the shaded bars are KRAS G13D mutants. **Panel B.** For 25 models ranked as in panel A, y axis shows RNA expression intensity [13], from top to bottom, for EGFR, EREG and AREG. RNA values are in log10 scale. **Panel C.** For 25 models ranked as in panel A, Y axis shows gene copy number per PICNIC [13]. The cetuximab treatment in mice was extensively described previously [13]. Briefly, when tumor volume reaches 100–150 mm3, the mice were grouped and subjected to either PBS or cetuximab (IP, weekly for 2 weeks, 1mg per mouse).

We were first interested in testing certain proposed expression biomarkers predictive of cetuximab response in CRC patients, including AREG and EREG expression [16, 18], using PDX based MCT datasets. We recently described a MCT using a cohort of 25 CRC-PDXs [7]. We examined the mRNA levels of EGFR/AREG/EREG genes, determined by transcriptome sequencing (RNA-seq) analysis. However, the data did not point to any apparent correlation with the response to cetuximab as measured by ^ΔT^/_ΔC_ (Figure 1). Copy numbers of EGFR/AREG/EREG genes, determined by Affymetrix SNP6.0 chip analysis, did not show correlation in the tested PDXs, either (Figure 1). These results are consistent with the clinical observations [19, 20].

### RAS pathway signature seems to describe correlation with cetuximab response in this cohort of CRC-PDXs

KRAS mutations are common in CRC, suggesting the close involvement of KRAS activity in CRC pathogenesis [7]. RAS gene mutations, *e.g*. KRAS and BRAF, have also been associated with resistance to cetuximab [7]. We therefore reasoned that gene expression profiles related to RAS pathway activity may have a certain relationship to cetuximab response. On the other hand, although the absolute role of each KRAS mutation allele is in question with regard to CRC response to cetuximab [7], the activation status of the RAS signaling pathway downstream of EGFR could still play a role in clinical response to anti-EGFR therapies [21]. By analyzing three previously generated gene lists for scoring RAS signaling activity per expression profiling, Loboda and colleagues described an empirically-derived gene expression signature of RAS pathway activity based on the relative expression levels of 147 genes [17], so called the “RAS pathway signature”. These authors showed that their signature score may be more predictive of RAS pathway activity than KRAS mutation status, and that seems to have some correlation with the efficacy of cetuximab in treated mCRC patients by retrospective analysis using a published clinical dataset [17, 18] (Supplementary Figure S1).

**Figure 2:**
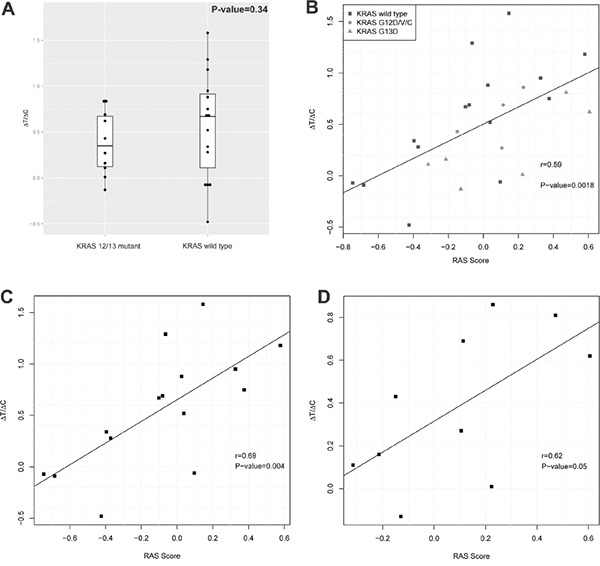
RAS pathway activity and cetuximab activity in a cohort of CRC-PDXs **A.** RAS signature score distribution in KRAS 12/13 mutants and KRAS 12/13 wild types. **B-D.** Correlation between RAS signature scores and tumor growth inhibition by cetuximab (^ΔT^/_ΔC_) in all 25 PDX models (B), in 15 KRAS 12/13 wild type PDX models (C), and in 10 KRAS 12/13 mutant PDX models (D).

We therefore assessed the RAS pathway signature scores [17] of the 25 CRC-PDXs from the cohort tested for cetuximab sensitivity in a MCT setting [7] (Table 1). We found that KRAS 12/13 mutants and wild types have similar pattern of RAS Signature scores, and there is no statistical difference between their means (Welch's two-sided t-test p-value=0.34, Figure 2A), suggesting that indeed KRAS 12/13 mutation status has insignificant correlation with RAS signature scores. When the cetuximab response was measured by ^ΔT^/_ΔC_, we observed a tight correlation between ^ΔT^/_ΔC_ and the RAS pathway signature scores with Pearson's correlation *r* = 0.59 and p-value=0.0018 (Figure 2B). Furthermore, we observed a correlation of *r* = 0.69 and p-value=0.004 for KRAS 12/13-wild type (Figure 2C), and *r* = 0. 62 and p-value= 0.05 for KRAS 12/13 mutants (Figure 2D). Such correlations on a completely independent cohort can hardly be explained by coincidence, and thus suggesting that the RAS pathway signature score, or RAS signaling for that matter, predicts the response of CRC-PDX to cetuximab. It is particularly interesting to note 6 of 15 KRAS-12/13-wild type PDXs have positive RAS pathway scores and are also associated with poor response (Table 1, Figure 2C). This implies that mechanisms other than KRAS 12/13-activating mutations can also up-regulate RAS signaling, consistent with previous report [7]. Similarly, 4 of 10 KRAS 12/13 mutants have negative scores and are associated with a certain degree of cetuximab sensitiveness.

**Table 1:** RAS Pathway Signature Scores and cetuximab sensitivity for 25 CRC-PDX models

Model ID	RAS Score	ΔT/ΔC	KRAS-12/13 Mutation
CR0047	0.105	0.27	G12C
CR0150	−0.149	0.43	G12D
CR0455	0.228	0.86	G12D
CR1554	0.113	0.69	G12V
CR0231	−0.129	−0.13	G13D
CR2520	0.223	0.01	G13D
CR0588	−0.315	0.11	G13D
CR0193	−0.214	0.16	G13D
CR2226	0.605	0.62	G13D
CR0012	0.472	0.81	G13D
CR2110	−0.424	−0.48	WT
CR2502	−0.684	−0.09	WT
CR0170	−0.746	−0.07	WT
CR0196	0.096	−0.06	WT
CR0560	−0.372	0.28	WT
CR0205	−0.396	0.34	WT
CR1530	0.038	0.52	WT
CR1519	−0.102	0.67	WT
CR0245	−0.081	0.69	WT
CR0004	0.375	0.75	WT
CR1574	0.025	0.88	WT
CR0029	0.326	0.95	WT
CR0146	0.578	1.18	WT
CR1744	−0.064	1.29	WT
CR0010	0.145	1.58	WT

## DISCUSSION

While molecular signatures per genetic alterations at DNA levels have met some success in predicting drug response, there seem few successes in identifying/validating signatures per epigenetic alterations, *e.g.* at mRNA expression levels. Our present study adds one even closer correlation of an independently derived RAS pathway signature [17], an mRNA signature, to the observed response to cetuximab per our MCT trial dataset [7], in addition to the previous observation of correlation in a clinical dataset [17]. In fact, our observed correlation in a MCT dataset is even stronger than that in the clinical dataset. Again, both datasets were derived from independent CRC cohorts, strongly suggesting the validity of this signature.

In repeating their calculations [17], we have confirmed that the correlation extends to the three Asian patients in the clinical dataset (Supplementary Figure S1). These observed correlations of the expression signature of RAS pathway to cetuximab response show the same assumed mechanism that downstream oncogenic signaling does not require an upstream signal via EGFR, thus cannot be suppressed by cetuximab, hence cetuximab resistant. The signature predicts both responders and non-responders, meaning it can be used to both exclude the non-responders (high scorers) and include responders (low scorers). In practice, it is possible that the RAS signature can be combined with oncogene mutation profiling [7] in the clinic for even better prediction. For example, patients screened with wild type KRAS gene can be further subjected to an mRNA expression profiling of these 147 genes in the RAS signature, and ones with lower RAS signature score have higher chance to respond to cetuximab treatment.

The RAS pathway signature scores as biomarkers can be readily obtained from biopsy samples from patients and used as exclusion/inclusion criteria for prospective clinical trials designed to validate it. Several pieces of evidence favor the potential success of this type of clinical trial. In particular, next generation sequencing (NGS), RNA-seq in this case, with its cost dramatically reduced recently, enables this 147-gene transcription signature readily practical in the clinic. Furthermore, a simpler signature and a companion diagnostic kit could also be developed.

^ΔT^/_ΔC_, or TGI, is commonly used in preclinical oncology to get pharmacology readouts. With the introduction of a MCT concept [7, 22–24], it is important to determine whether such readouts best predict clinical readouts. Investigations to address this by evaluating different “clinically relevant” readouts in a variety of MCT settings have been attempted. We are also actively exploring this by examining and comparing different candidate MCT endpoints at present on a number of MCT datasets, including this one, hoping to identify the pros and cons of different endpoints and also the best ones for certain applications.

## MATERIALS AND METHODS

### RAS pathway signature score calculation

The scores were calculated independently in two gene expression datasets: 25 PDX samples and 68 samples with Affymetrix U133A 2.0 array data from clinical dataset GSE5851 (the latter recapitulates a calculation originally shown by Laboda et al [17]). The signature described contains 105 genes in the ‘Up’ set and 42 genes in the ‘Down’ set, for which we could map 97 genes/and 32 genes respectively on RNA-seq data, and 90 genes and 33 genes respectively on the HG-U133A 2.0 array. All gene expression was normalized to have zero mean and unit variance. The RAS scores were calculated by subtracting the average expression of ‘Down’ genes from that of the ‘Up’ genes. A higher score indicates stronger RAS pathway activation.

## SUPPLEMENTARY MATERIALS FIGURE



## References

[1] Ciardiello F, Tortora G (2008). EGFR antagonists in cancer treatment. N Engl J Med.

[2] De Roock W, Claes B, Bernasconi D, De Schutter J, Biesmans B, Fountzilas G, Kalogeras KT, Kotoula V, Papamichael D, Laurent-Puig P, Penault-Llorca F, Rougier P, Vincenzi B (2010). Effects of KRAS, BRAF, NRAS, and PIK3CA mutations on the efficacy of cetuximab plus chemotherapy in chemotherapy-refractory metastatic colorectal cancer: a retrospective consortium analysis. Lancet Oncol.

[3] De Roock W, De Vriendt V, Normanno N, Ciardiello F, Tejpar S (2011). KRAS, BRAF, PIK3CA, and PTEN mutations: implications for targeted therapies in metastatic colorectal cancer. Lancet Oncol.

[4] Allegra CJ, Jessup JM, Somerfield MR, Hamilton SR, Hammond EH, Hayes DF, McAllister PK, Morton RF, Schilsky RL (2009). American Society of Clinical Oncology provisional clinical opinion: testing for KRAS gene mutations in patients with metastatic colorectal carcinoma to predict response to anti-epidermal growth factor receptor monoclonal antibody therapy. J Clin Oncol.

[5] Cunningham D, Humblet Y, Siena S, Khayat D, Bleiberg H, Santoro A, Bets D, Mueser M, Harstrick A, Verslype C, Chau I, Van Cutsem E (2004). Cetuximab monotherapy and cetuximab plus irinotecan in irinotecan-refractory metastatic colorectal cancer. N Engl J Med.

[6] De Roock W, Jonker DJ, Di Nicolantonio F, Sartore-Bianchi A, Tu D, Siena S, Lamba S, Arena S, Frattini M, Piessevaux H, Van Cutsem E, O'Callaghan CJ, Khambata-Ford S (2010). Association of KRAS p. G13D mutation with outcome in patients with chemotherapy-refractory metastatic colorectal cancer treated with cetuximab. JAMA.

[7] Chen D, Huang X, Cai J, Guo S, Qian W, Wery JP, Li QX (2015). A set of defined oncogenic mutation alleles seems to better predict the response to cetuximab in CRC patient-derived xenograft than KRAS 12/13 mutations. Oncotarget.

[8] Bertotti A, Papp E, Jones S, Adleff V, Anagnostou V, Lupo B, Sausen M, Phallen J, Hruban CA, Tokheim C, Niknafs N, Nesselbush M, Lytle K, Sassi F, Cottino F, Migliardi G (2015). The genomic landscape of response to EGFR blockade in colorectal cancer. Nature.

[9] Di Nicolantonio F, Martini M, Molinari F, Sartore-Bianchi A, Arena S, Saletti P, De Dosso S, Mazzucchelli L, Frattini M, Siena S, Bardelli A (2008). Wild-type BRAF is required for response to panitumumab or cetuximab in metastatic colorectal cancer. J Clin Oncol.

[10] Yonesaka K, Zejnullahu K, Okamoto I, Satoh T, Cappuzzo F, Souglakos J, Ercan D, Rogers A, Roncalli M, Takeda M, Fujisaka Y, Philips J, Shimizu T (2011). Activation of ERBB2 signaling causes resistance to the EGFR-directed therapeutic antibody cetuximab. Sci Transl Med.

[11] Lievre A, Bachet JB, Boige V, Cayre A, Le Corre D, Buc E, Ychou M, Bouche O, Landi B, Louvet C, Andre T, Bibeau F, Diebold MD (2008). KRAS mutations as an independent prognostic factor in patients with advanced colorectal cancer treated with cetuximab. J Clin Oncol.

[12] Sequist LV, Soria JC, Goldman JW, Wakelee HA, Gadgeel SM, Varga A, Papadimitrakopoulou V, Solomon BJ, Oxnard GR, Dziadziuszko R, Aisner DL, Doebele RC, Galasso C (2015). Rociletinib in EGFR-mutated non-small-cell lung cancer. N Engl J Med.

[13] Yang M, Shan B, Li Q, Song X, Cai J, Deng J, Zhang L, Du Z, Lu J, Chen T, Wery JP, Chen Y (2013). Overcoming erlotinib resistance with tailored treatment regimen in patient-derived xenografts from naive Asian NSCLC patients. Int J Cancer.

[14] Yasuda H, Kobayashi S, Costa DB (2012). EGFR exon 20 insertion mutations in non-small-cell lung cancer: preclinical data and clinical implications. Lancet Oncol.

[15] Koivunen JP, Mermel C, Zejnullahu K, Murphy C, Lifshits E, Holmes AJ, Choi HG, Kim J, Chiang D, Thomas R, Lee J, Richards WG, Sugarbaker DJ (2008). EML4-ALK fusion gene and efficacy of an ALK kinase inhibitor in lung cancer. Clin Cancer Res.

[16] Jacobs B, De Roock W, Piessevaux H, Van Oirbeek R, Biesmans B, De Schutter J, Fieuws S, Vandesompele J, Peeters M, Van Laethem JL, Humblet Y, Penault-Llorca F, De Hertogh G (2009). Amphiregulin and epiregulin mRNA expression in primary tumors predicts outcome in metastatic colorectal cancer treated with cetuximab. J Clin Oncol.

[17] Loboda A, Nebozhyn M, Klinghoffer R, Frazier J, Chastain M, Arthur W, Roberts B, Zhang T, Chenard M, Haines B, Andersen J, Nagashima K, Paweletz C (2010). A gene expression signature of RAS pathway dependence predicts response to PI3K and RAS pathway inhibitors and expands the population of RAS pathway activated tumors. BMC Med Genomics.

[18] Khambata-Ford S, Garrett CR, Meropol NJ, Basik M, Harbison CT, Wu S, Wong TW, Huang X, Takimoto CH, Godwin AK, Tan BR, Krishnamurthi SS, Burris HA (2007). Expression of epiregulin and amphiregulin and K-ras mutation status predict disease control in metastatic colorectal cancer patients treated with cetuximab. J Clin Oncol.

[19] Lenz HJ, Van Cutsem E, Khambata-Ford S, Mayer RJ, Gold P, Stella P, Mirtsching B, Cohn AL, Pippas AW, Azarnia N, Tsuchihashi Z, Mauro DJ, Rowinsky EK (2006). Multicenter phase II and translational study of cetuximab in metastatic colorectal carcinoma refractory to irinotecan, oxaliplatin, and fluoropyrimidines. J Clin Oncol.

[20] Chung KY, Shia J, Kemeny NE, Shah M, Schwartz GK, Tse A, Hamilton A, Pan D, Schrag D, Schwartz L, Klimstra DS, Fridman D, Kelsen DP, Saltz LB (2005). Cetuximab shows activity in colorectal cancer patients with tumors that do not express the epidermal growth factor receptor by immunohistochemistry. J Clin Oncol.

[21] Young A, Lyons J, Miller AL, Phan VT, Alarcon IR, McCormick F (2009). Ras signaling and therapies. Adv Cancer Res.

[22] Gao H, Korn JM, Ferretti S, Monahan JE, Wang Y, Singh M, Zhang C, Schnell C, Yang G, Zhang Y, Balbin OA, Barbe S, Cai H (2015). High-throughput screening using patient-derived tumor xenografts to predict clinical trial drug response. Nat Med.

[23] Julien S, Merino-Trigo A, Lacroix L, Pocard M, Goere D, Mariani P, Landron S, Bigot L, Nemati F, Dartigues P, Weiswald LB, Lantuas D, Morgand L (2012). Characterization of a large panel of patient-derived tumor xenografts representing the clinical heterogeneity of human colorectal cancer. Clin Cancer Res.

[24] Zhang L, Yang J, Cai J, Song X, Deng J, Huang X, Chen D, Yang M, Wery JP, Li S, Wu A, Li Z, Liu Y, Chen Y, Li Q, Ji J (2013). A subset of gastric cancers with EGFR amplification and overexpression respond to cetuximab therapy. Sci Rep.

