# Supplementary material for: Cetuximab response in CRC patient-derived xenografts seems predicted by an expression based RAS pathway signature
Source: Oncotarget. 2016 Jul 8;7(31):50575–81. doi: 10.18632/oncotarget.10499 (PMC5226604; doi:10.18632/oncotarget.10499)
Supplement: Supplementary file 1 [file oncotarget-07-50575-s001.pdf]

Cetuximab response in CRC patient-derived xenografts seems predicted by an expression based RAS pathway signature

SUPPLEMENTARY FIGURE

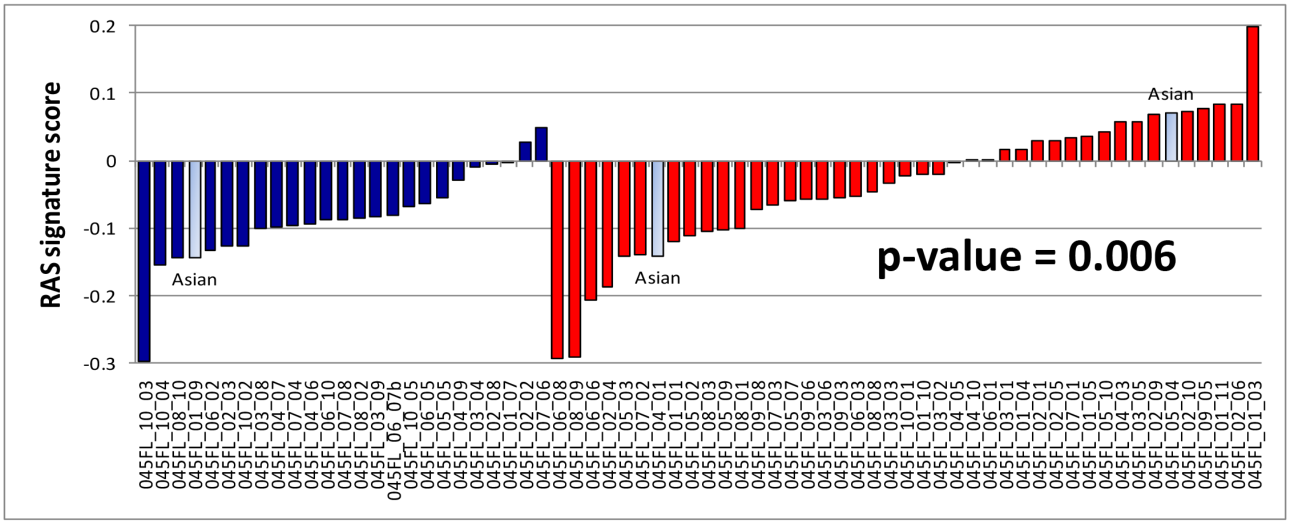

Supplementary Figure S1: RAS pathway activity and cetuximab sensitivity in clinical trial CA225-045. Waterfall plot of RAS signature scores in the patients who experienced disease control (25; blue) or progressive disease (43; red) after cetuximab therapy. Results for three patients of Asian origin are indicated. The Y axis shows RAS signature score relative to the mean in this dataset. Scores were derived using microarray data from GSE5851 in the GEO database.
